# Ablation of Post Transplant Atrial Flutter and Pseudo-fibrillation Using Magnetic Navigation via a Superior Approach

**DOI:** 10.1016/s0972-6292(16)30546-0

**Published:** 2012-09-01

**Authors:** Roderick Tung, Kalyanam Shivkumar, Ravi Mandapati

**Affiliations:** UCLA Cardiac Arrhythmia Center, David Geffen School of Medicine at UCLA, Los Angeles, CA; Loma Linda University Medical Center, Division of Pediatric Cardiology, Loma Linda, CA

**Keywords:** pseudo-fibrillation, magnetic navigation, post transplant

## Abstract

Ablation of cavotricuspid ishtmus flutter and atrial tachycardia in a complex substrate has never been reported using remote navigation via superior approach. Venous access was obtained via right internal jugular for ablation and left subclavian for duodecapolar catheter placement into the coronary sinus. In a posttransplant patient presenting with both regular and irregular tachycardia, both cavotricuspid isthmus flutter in the donor and atrial tachycardia in the recipient was mapped using a two catheter approach. Successful ablation of typical atrial flutter and anastomotic block was achieved. This is the first report of successful ablation of cavotricuspid isthmus flutter and posttransplant atrial tachycardia using magnetic navigation via superior approach. Using only two catheters, this approach is logical and feasible in complex substrates with interrupted inferior venous access.

## Case

A 17-year-old man with a history of dilated cardiomyopathy requiring orthotopic heart transplant at 5 months of age presents with multiple recurrences of atrial flutter and fibrillation. The transplant performed utilized an atrio-atrial anastomosis between recipient and donor. His posttransplant course was complicated by low grade rejection at the age of 3 years, but since then has remained stable on immunosuppressive therapy in his adult life. Most recent ejection fraction was estimated at 55%. His sixth episode of atrial arrhythmia was consistent with typical counterclockwise atrial flutter with cycle length of 220ms, which was treated with cardioversion. Two episodes of atrial fibrillation were seen additionally. Both femoral veins were known to be totally occluded due to chronic proximal deep venous thromboses bilaterally and he was referred for mapping and ablation of atrial flutter via a superior venous approach using magnetic navigation.

Access was obtained with an 8F locking sheath via left subclavian vein for widely spaced duodecapolar catheter and a 9F sheath via the right internal jugular vein for mapping ablation. A 3.5mm irrigated Navistar ablation catheter (Biosense-Webster, Diamond Bar, CA) was used. The duodecapolar was placed into the coronary sinus with the proximal bipoles on the lateral wall of the right atrium. A right atrial contrast injection was performed and registered with the mapping system (CARTO RMT, Biosense-Webster). An electroanatomic map was created using magnetic navigation (Stereotaxis, St. Louis, MO) with CARTO RMT in the baseline junctional rhythm. Isuprel was given intravenously to promote sinus tachycardia and to expedite atrial mapping. The donor heart demonstrated normal bipolar amplitude without evidence of scar and the recipient was noted to have diffuse areas of low voltage.

**Figure 1:**
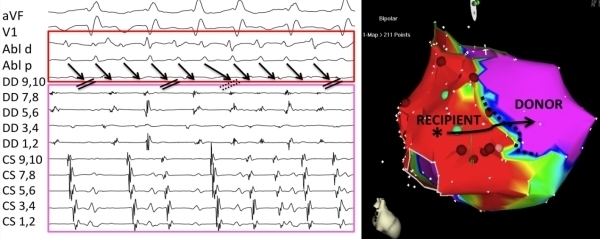
Recipient atrial tachycardia with variable conduction to donor appearing like atrial fibrillation. Wenckebach periodicity is seen as well prior to block of 1:1 conduction. (dashed double line)

**Figure 2:**
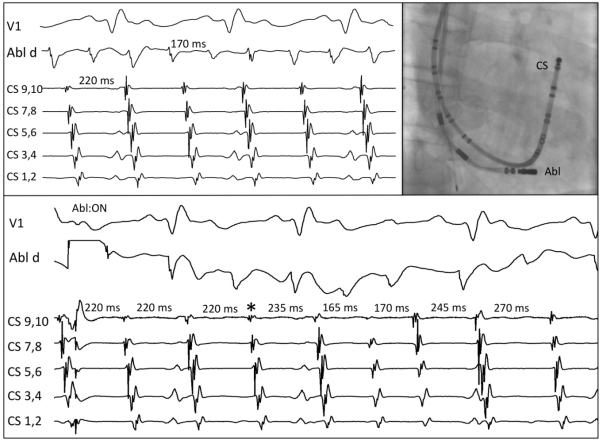
Two tachycardias dissociated with different cycle lengths, with Abl d in recipient atrium (upper, left). Ablation in the donor cavotricuspid isthmus (upper right, RAO view) terminates typical atrial flutter (asterisk) and variable conduction of atrial tachycardia from the recipient is seen (lower).

Atrial flutter was induced with atrial burst pacing at 200 ms, but was nonsustained. Atrial fibrillation was induced with burst pacing down to 150ms (Figure 1). On closer examination, the irregular rhythm induced demonstrated a singular and constant activation pattern from lateral wall to septum with proximal to distal coronary sinus activation. The mapping catheter was placed in the recipient heart and an atrial tachycardia with cycle length of 150-180 ms was seen. Recipient atrial tachycardia with variable conduction to the donor heart mimicked atrial fibrillation (pseudo-atrial fibrillation), which was clinically documented. Wenckebach periodicity was noted when 1:1 conduction progressed to block. Shortly after, typical counterclockwise atrial flutter was spontaneously initiated in the donor heart (TCL 220 ms). Entrainment via the coronary sinus catheter from the medial isthmus demonstrated a PPI-TCL of 25 ms. The isthmus conduction time was measured at 70 ms during cavotricuspid isthmus flutter. Dissociation of the two tachycardias in recipient and donor was demonstrated. (Figure 2 upper)

Linear ablation was delivered in the cavotricuspid isthmus via the right internal jugular access (35W, T 35-42). Due to a superior approach, a continuous drag could not be applied, but rather point-by-point ablation with manipulation after each ablation. During the fourth radiofrequency application, termination of cavotricuspid isthmus flutter occurred, manifest by a change to passive variable conduction of the recipient atrial tachycardia (Figure 2 lower, asterisk).

**Figure 3:**
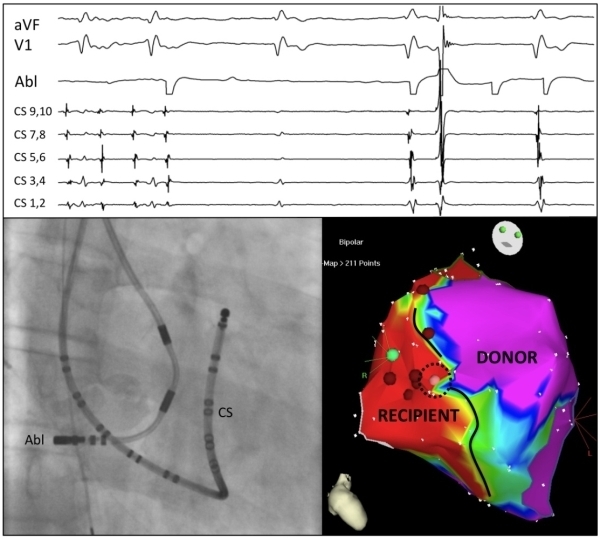
Completion of anastomotic conduction block along septal suture line (pink ablation lesion in dashed conduction gap). Junctional rhythm after a pause is seen in the donor during ablation. Atrial tachycardia persisted in the recipient when the ablation catheter was repositioned after ablation.

The recipient tachycardia was then targeted. Overdrive pacing within the scar demonstrated a postpacing interval of 20-30 ms at multiple locations. An activation map created with CARTO demonstrated multiple regions of "early-meets-late". Ablation at fractionated and split potential location within the scar failed to terminate tachycardia. Several electrograms that spanned over half of the cycle length were in regions of phrenic capture (20mA, 2 ms). Therefore, ablation was focused on completing anastomotic block between the recipient to donor. Ablation along the septal anastomotic line from superior vena cava to inferior vena cava failed to alter the conduction of atrial tachycardia to the recipient heart. A single ablation at the low posterolateral anastomosis resulted in conduction block out of the recipient heart, with resumption of junctional rhythm after a long pause. (Figure 3) Atrial tachycardia persisted in the recipient heart. Multiple consolidating radiofrequency applications were performed in this region. A synchronized cardioversion was delivered to the recipient heart with restoration to sinus rhythm as the mechanism of the recipient atrial tachycardia could not be determined by mapping in the right atrium. The mechanism was thought to be intrascar microreentry or an arrhythmia originated from the pulmonary veins or the left atrial side of the recipient heart. Dissociation of recipient sinus and donor junctional rhythms was seen. Bidirectional block (150ms) across the cavotricuspid isthmus was also demonstrated. The total case duration was 8.5 hours with a fluoroscopy time of 30 minutes.

## Discussion

To our knowledge, this is the first report of magnetically-navigated cavotricuspid isthmus and scar-mediated atrial tachycardia ablation using a superior venous approach. While magnetic navigation has been shown to expedite procedure times with comparable efficacy compared to manual strategy, results for cavotricuspid isthmus flutter have been variable.[1] Ablation via internal jugular and subclavian access has been reported for AV node reentry, typical atrial flutter, and atrial fibrillation. [2,3]

The cavotricuspid isthmus is a particularly challenging region to ablate from a superior approach as it does not follow the natural curve to "drag" toward the inferior vena cava and requires the operator to work at the head of the table. When working at the head of the table, a natural resting of position of the catheter on the patients legs makes manipulation more difficult. For this reason, we believe that magnetic navigation facilitated a controlled linear movement along the cavotricuspid isthmus from the superior approach. Additionally, radiation shielding is more difficult as well as the ability to view monitors, resulting in increased exposure to the operator. This case demonstrates the feasibility of a combined superior approach with magnetic guidance in a complex substrate, whereby access and contact in all regions of the right atrium was adequate for mapping and ablation. Given the prolonged duration of the procedure, the fluoroscopy time was relatively lower than cases performed at our institution of comparable complexity (atrial fibrillation-45 minutes, ventricular tachycardia 60 minutes).

Typical cavotricuspid isthmus flutter remains the commonest atrial arrhythmia post transplant.[4] Complete conduction block, Wenckebach, and 2:1 conduction across suture lines have been previously reported.[5] The finding of variable conduction block of atrial tachycardia across atrio-atrial anastomosis appearing as pseudo-fibrillation is unique. Atrial tachycardia with variable recipient to donor conduction should be considered in the differential diagnosis of an irregular rhythm in post transplant patients.
